# Case of Uric Acid Calculus, Which Appeared to Have Been Dissolved in the Pelvis of the Left Kidney by Alkaline Remedies

**Published:** 1884-10

**Authors:** Harlan N. Orton

**Affiliations:** Minneapolis, Minn.


### ARTICLE IV.

### CASE OF URIC ACID CALCULUS, WHICH APPEARED TO HAVE BEEN DISSOLVED IN THE PELVIS OF THE LEFT KIDNEY, BY ALKALINE REMEDIES. *By* HARLAN N. ORTON, M. D., *Minneapolis, Minn.*

Having arrived at your diagnosis in cases of calculi in the kidney, and having determined in your own mind that a stone exists, the question arises, What course are you to pursue?

The best authorities assert that "the medical treatment in these cases is unsatisfactory, and can at the best be only palliative." Now, as I do not belong to the so-called "school of medical skepticism," I have come to the conclusion from the result obtained in a case (the history of which I am about to relate) that it would be just as well to first try what remedies will accomplish before we resort to the knife.

*Case.*—James G., age 45 years; large, fleshy man, of a gouty diathesis. I was called to see him first, August 2d, in the morning; found him suffering from a severe attack of nephritic colic; said he had the first attack of this kind while in the Union army, about 1862, and a recurrence of the same trouble on an average of about once in six months ever since, passing a small calculus at each time, one of which he had saved and I took possession of. I concluded, from the history he gave me of previous attacks, that this one would pass off about the same, so prescribed Dover's powder and applications of hot cloths to relieve pain, etc. Called again in the evening; found him resting quite easy; said the paroxysms of pain would come on about every two or three hours and last for half an hour, when it seemed as if the stone would work back into the pelvis of the kidney, and then he would have relief for a while.

August 3d (morning), condition about the same, except the pain would be a little more severe at times.

I quote his own words, "Doctor, I am in for it this time. That stone is too large to ever pass down into the bladder, unless you can give me some medicine to dissolve off part of it in the kidney."

The question came to my mind, Is a doctor justified in folding his arms and waiting, in such a case as this, in hopes that the calculus might become encysted? I think not, any how not until he has tried the solvent properties of the different remedies.

I analyzed the calculus that he had passed in one of his previous attacks, and found it to be composed of uric acid principally, and I had good reason to suppose that the one in his kidney at this time was of the same composition. The urine gave a marked acid reaction.

After a consultation with a medical friend of mine, we concluded to put the patient on alkaline remedies—potassii acetas, gr. X, lithii carbonas, gr. iij, every hour, largely diluted with distilled water. In about twenty-four hours after commencement of this treatment, he began to pass "*red gravel*" with his urine; soon after he experienced instant relief from all pain—said he could feel the stone drop into the bladder. At 5 P. M. same day (August 4th), passed a calculus with his urine, one-half inch long by one-quarter inch in diameter, composed of uric acid, quite soft—would almost crumble between the fingers while handling it. The concretions that he passed altogether would about fill a tablespoon. His urine soon cleared up, and he has had no trouble since. The result I obtained in this case is certainly in favor of this method of treatment.

